# In Silico Ultrafast Nonlinear Spectroscopy Meets Experiments: The Case of Perylene Bisimide Dye

**DOI:** 10.1021/acs.jctc.1c00570

**Published:** 2021-10-22

**Authors:** Francesco Segatta, Mattia Russo, Daniel R. Nascimento, Davide Presti, Francesco Rigodanza, Artur Nenov, Andrea Bonvicini, Alberto Arcioni, Shaul Mukamel, Margherita Maiuri, Luca Muccioli, Niranjan Govind, Giulio Cerullo, Marco Garavelli

**Affiliations:** † Dipartimento di Chimica Industriale “Toso Montanari”, Università di Bologna, Viale del Risorgimento 4, Bologna I-40136, Italy; ‡ IFN-CNR, Dipartimento di Fisica, Politecnico di Milano, P. Leonardo da Vinci 32, Milan I-20133, Italy; § Physical and Computational Sciences Directorate, Pacific Northwest National Laboratory, Richland, Washington 99352, United States; ∥ Dipartimento di Scienze Chimiche, Università degli studi di Padova, Via F. Marzolo, Padova I-35131, Italy; ⊥ Department of Chemistry and Department of Physics and Astronomy, University of California, Irvine, California 92697, United States; # Department of Chemistry, The University of Memphis, Memphis, Tennessee 38152, United States

## Abstract

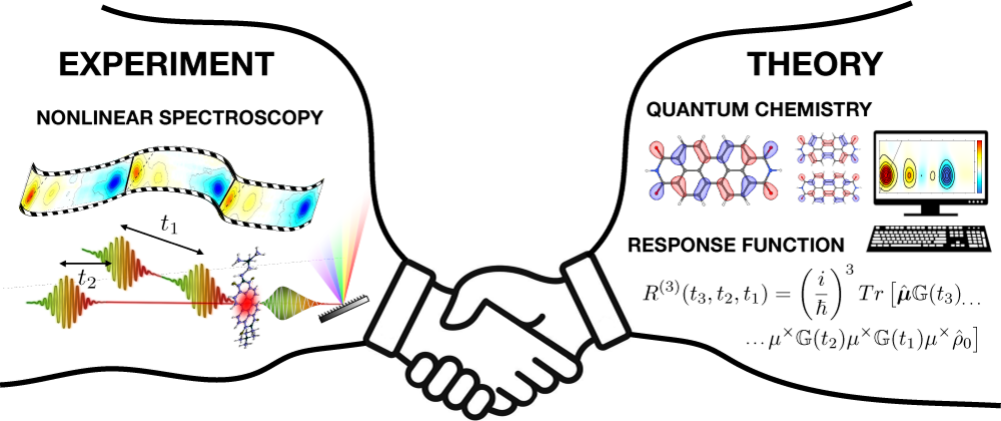

Spectroscopy simulations are of paramount importance for the interpretation of experimental electronic spectra, the disentangling of overlapping spectral features, and the tracing of the microscopic origin of the observed signals. Linear and nonlinear simulations are based on the results drawn from electronic structure calculations that provide the necessary parameterization of the molecular systems probed by light. Here, we investigate the applicability of excited-state properties obtained from linear-response time-dependent density functional theory (TDDFT) in the description of nonlinear spectra by employing the pseudowavefunction approach and compare them with benchmarks from highly accurate RASSCF/RASPT2 calculations and with high temporal resolution experimental results. As a test case, we consider the prediction of femtosecond transient absorption and two-dimensional electronic spectroscopy of a perylene bisimide dye in solution. We find that experimental signals are well reproduced by both theoretical approaches, showing that the computationally cheaper TDDFT can be a suitable option for the simulation of nonlinear spectroscopy of molecular systems that are too large to be treated with higher-level RASSCF/RASPT2 methods.

## 1 Introduction

Nonlinear electronic spectroscopy with temporal resolution down to the femtosecond (fs) time scale has become an increasingly important tool for studying the structure and dynamics of molecular systems, ranging from single molecules to large molecular aggregates.^1−5^ In typical nonlinear spectroscopy experiments, a sequence of laser pulses, with controlled time delays and phases, is focused on the sample to bring it out of equilibrium and subsequently monitor its evolution. Specifically, in transient absorption (TA) and two-dimensional electronic spectroscopy (2DES), two interactions with the pump laser field prepare the system in an initial excited state. The relaxation back to equilibrium is monitored by illuminating the system with a delayed pulse (i.e., the probe) and scanning the pump–probe delay. The molecular dynamics is captured indirectly by means of the spectral dynamics of ground-state bleaching (GSB) of the photoexcited transition, stimulated emission (SE) from the prepared excited state to the ground state, and photoinduced absorption to high-lying states. With respect to TA, 2DES provides resolution not only in the detection but also in the excitation frequency axis, delivering for each value of the pump–probe delay a 2D map correlating the excitation and detection frequencies.

Despite the continuous technical progress in ultrafast spectroscopy, the direct interpretation of the measured transient spectra, often congested by many overlapping contributions of different origins, may be hard and rarely univocal. The integration of experimental and computational approaches has therefore become of paramount importance in the analysis and interpretation of transient optical spectroscopy, spanning the infrared–vis–UV spectral regimes,^5−9^ enabling the disentanglement of all measured features, and obtaining a detailed and reliable description of the system dynamics.

The reliability of simulations to support the interpretation of experimental signals and furnish a microscopic understanding of the studied molecular systems is grounded on an accurate description of the molecular electronic structure and a simple and robust model for the spectroscopic technique, able to provide connection with the experimentally measured observables.^10^ Accurate electronic structure calculations can be obtained from a wide range of methodologies, some of which are readily available in standard quantum chemistry (QC) packages. In theoretical nonlinear spectroscopy studies, the electronic structure is usually obtained from multireference methods, such as the restricted active space self-consistent-field (RASSCF) and its second-order perturbation theory variant (RASPT2) due to their universal and potentially accurate treatment of highly excited states required for simulating TA/2DES features.^11^ Indeed, a well-designed active space can describe simultaneously and on an equal footing states of different characters, such as covalent and ionic (e.g., charge transfer), and single and multiple excitations. For instance, the RASSCF/RASPT2 level of theory has been utilized recently to simulate fully ab initio TA spectra of the ultrafast nonadiabatic dynamics of several organic molecules—azobenzene,^12^ pyrene,^13^ uridine and thymidine,^14^ and thiouracil^8,15,16^—in remarkable agreement with the experiment.

The high computational cost of multireference methods limits their applicability to relatively small active spaces. An inexpensive alternative for the treatment of molecular systems is the linear-response formulation of time-dependent density functional theory (TDDFT). Although in its original formulation TDDFT describes only excitations from the ground state, and it is thus unsuitable to the description of nonlinear spectroscopy, it can be a valuable tool in cases where the initial excited state is predominantly single determinant. In this case, an initially excited state can be prepared enforcing the singly excited configuration with the maximum-overlap method^17,18^ and then can be used as a reference for the TDDFT computation, eventually yielding the desired TA features.^19−21^

In this work, we explore the applicability of excited states obtained from TDDFT in the description of a nonlinear optical response by employing the pseudowavefunction approach^22^ and provide benchmarks against the highly accurate RASSCF/RASPT2 method. Both computational approaches are compared to the experimental results obtained by high time-resolution TA and 2DES spectroscopy on the bis-cationic *N*,*N*′-bis(2-(trimethylammonium)ethylene)perylene-3,4,9,10-tetracarboxylic acid bisimide dye, hereafter labeled perylene bisimide (PBI)-(R^+^)_2_ (R^+^ being the 2-(trimethylammonium)ethylene side chain). PBI derivatives have been proposed and tested for advanced organic optoelectronic applications, such as organic semiconductors and solar cells, as well as for photoredox catalysis (e.g., water splitting)^23^ and are also studied as an archetypal functional supramolecular material^24^ and show pronounced chromonic self-ordering properties.^24,25^ Moreover, the planar, highly symmetric structure of the PBI core gives rise to neat spectral properties, such as a vibrationally resolved linear absorption (LA) spectrum from a single bright electronic state in the visible region,^26^ with a long lifetime and only few characteristic excited-state absorption (ESA) bands. This makes PBIs ideally suited test cases for the comparison of simulated and experimental spectroscopy. The excellent agreement of the simulations with the experimental results proves the power of in silico ultrafast spectroscopy in interpreting and guiding experimental studies.

This paper is organized as follows: first a description of the theoretical and experimental methods used in this work is given, highlighting the novelty and reliability of the TDDFT approach in the context of nonlinear electronic spectroscopy; the presentation of the results and their detailed discussion follows, with a comparison of the simulated and measured PBI linear and nonlinear spectra, comprising TA and 2DES time-resolved spectra, and analysis of the detected coherent vibrational modes; the concluding paragraph summarizes the presented material and outlines possible routes for further developments.

## 2 Theoretical Methods

**Figure 1:**
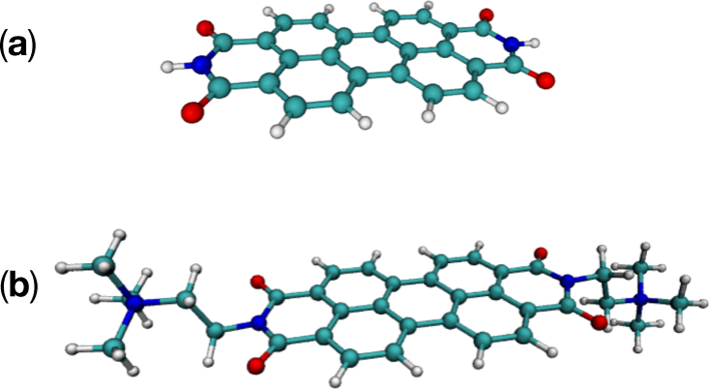
(a) PBI and (b) PBI-(R^+^)_2_ molecular structures. R^+^ indicates each of the two 2-(trimethylammonium)ethylene positively charged side chains.

We first show how QC ingredients enter in the simulation of spectroscopy, highlighting the electronic structure calculations that need to be performed to access the molecular manifold of states. We introduce the extended linear-response TDDFT approach, capable of providing dipole moments and excited-state dipole couplings. RASSCF/RASPT2 computations are then presented. Test calculations were performed on both the neutral PBI and the PBI-(R^+^)_2_ dication, whose molecular structures are shown in Figure 1. PBI-(R^+^)_2_ is the system synthesized and investigated experimentally. The neutral PBI, characterized by a higher symmetry with respect to the cationic species (*D*_2*h*_ and *C*_2*h*_, respectively) is computationally more tractable, especially for energy-gradient calculations. Since the electronic structure of both molecules is nearly identical (as discussed in Sections S3–S5 of the Supporting Information), QC calculations and spectral simulations at both the RASSCF/RASPT2 and TDDFT levels of theory were performed on the neutral PBI.

### 2.1 Spectroscopy Simulations Including Harmonic Vibrations

The response function formalism is the natural framework in which analytic expressions for the description of linear and nonlinear spectroscopy can be written, also including harmonic vibrations (via cumulant expansion of Gaussian fluctuations).^27,28^ A detailed presentation of the spectroscopic methods employed herein is shown in ref (10). Concisely, one considers a molecular system described by a time-dependent Hamiltonian
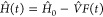
1where *Ĥ*_0_ is the field-free molecular Hamiltonian and *V̂* is the operator that couples an external laser field of the form *F*(*t*) with the system. Within the electric dipole approximation, valid when the wavelength of the external field is long compared to the molecular dimensions, the field-molecule coupling takes place via the electric dipole operator **μ̂**.

The response of an observable Ω(*t*) to the external field *F*(*t*) is given by^28^
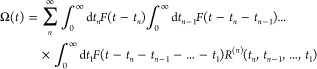
2where *R*^(*n*)^(*t*_*n*_,*t*_*n*–1_,...,*t*_1_) is the *n*th-order response function defined as
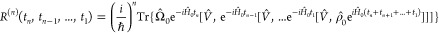
3where Ω̂_0_ and ρ̂_0_ correspond to the operators for the observable Ω and the density ρ at equilibrium, respectively.

In general, the choice of the observable and the form of the perturbation are associated with a given spectroscopy and thus are predefined. Hence, all that remains is to compute the response function, which consists of a sequence of field-molecule interactions (described by *V̂*), followed by field-free propagations of the molecular electronic and nuclear degrees of freedom, driven by *Ĥ*_0_.

Since in the present work we focus on the first- and third-order responses to the field interaction (for linear and nonlinear spectroscopy simulations, respectively), *R*^(1)^(*t*_1_) and *R*^(3)^(*t*_3_,*t*_2_,*t*_1_) are explicitly expressed below as
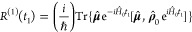
4
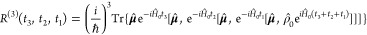
5where Ω̂_0_ and *V̂* have both been replaced by **μ̂**.

The electronic-nuclear coupling, which has the definition of *Ĥ*_0_, is modeled via the displaced harmonic oscillator (DHO), which assumes the potential energy surfaces of the electronic states in the space of nuclear degrees of freedom to be identical harmonic wells with different equilibrium positions (i.e., displaced along the nuclear coordinates). The Condon approximation is also invoked, in which the dipole moment operator is independent of the nuclear coordinates. Furthermore, we assume that the states of interest are long-lived with respect to the time scale of the experiment. These approximations are appropriate for the case of PBI as (a) it is a rigid molecule, for which both the DHO and the Condon approximation are well justified, and (b) it is experimentally proven that the PBI bright S_1_ state does not show any fast deactivation dynamics (it decays to the ground state on a several nanoseconds time scale), making the long-lifetime assumption sensible. In passing, we note that adaptation of the present modeling to less rigid or even flexible molecules has been reported in the literature.^26−103^

The necessary information for building *Ĥ*_0_ within the DHO model was obtained from either RASSCF/RASPT2 or TDDFT computations. In both cases, the relevant quantities are as follows:

- excited-state energies;
- transition dipole moments between all states;
- excited-state energy gradients;
- ground-state normal modes and frequencies.

The excited-state energy gradients comprise the gradients of the states prepared by the pump pulse (S_1_ in the case of PBI), as well as those of higher-lying excited states that are contained in the spectral window of interest (defined by the experiment), and exhibit an oscillator strength from S_1_ above a certain threshold (chosen as 0.02).

The excited-state energy gradients and ground-state normal modes and frequencies are used to construct vibrational spectral densities that eventually encode the intramolecular part of the electronic-nuclear coupling. Coupling between the electronic states and the environment (e.g., the solvent) is introduced by a phenomenological overdamped Brownian oscillator (OBO) spectral density, whose functional form is given by
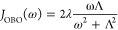
6where λ is the solute–solvent interaction strength (or, in other words, the solvent-induced reorganization energy) and Λ^–1^ is the so-called dephasing (or correlation) time. These two parameters are typically fitted to the experimental data. Here, we employed λ = 240 cm^–1^ and Λ^–1^ = 40 fs.

All the spectra presented here have been computed employing the sum-over-states approach implemented in the Spectron software package for the simulation of optical spectroscopy.^27^ The information flow from the QC codes to Spectron, as well as the analysis and plotting of the obtained spectroscopic data, is managed through the recently developed iSPECTRON interface.^10^ Reference (10) also contains details about Spectron functioning, inputs, and parameters.

### 2.2 Pseudowavefunction Approach to TDDFT

By considering the response in the density to a density perturbation (the density–density response), one can obtain a hierarchy of *n*th-order corrections to the density response that can be used in the computation of transition properties. Such an approach, when applied to density functional theory (DFT), yields the well-known equations for linear-response TDDFT, as well as their higher-order analogues.^29^

TDDFT gives access to a perturbed pseudowavefunction^30^ that may be employed in the computation of transition moments between the ground and excited states.^29,31^ However, in order to simulate nonlinear spectroscopy, transition moments between excited states are required as well. These transition moments arise naturally as part of the quadratic response and may be obtained as its double residue. Nonetheless, it has been demonstrated that for approximate theories like TDDFT, the quadratic response contains unphysical poles whenever the transition energy between two excited states matches the excitation energy of any other state.^32−34^ These unphysical features may be tamed by employing a complex polarization propagator approach at the expense of losing valuable information regarding the nature of the states participating in the absorption process, but the unphysical poles are still present and give rise to resonances that have no equivalent in exact theory. Hence, the quadratic-response formalism has to be used with caution, and furthermore, its additional computational cost imposes limitations on the size of the systems that can be studied.^35^

An alternative that has been widely employed in the computation of derivative couplings^22,30,36,37^ and transition moments between excited states^21,38−40^ is to completely neglect dynamical orbital relaxation effects and use the amplitudes obtained from linear-response TDDFT to construct excited-state couplings and transition moments including up to single excitations.

In the TDDFT pseudowavefunction formalism,^30^ an excited-state pair {|*K*⟩, |*L*⟩} is defined as
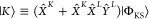
7and
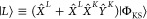
8where |Φ_KS_⟩ is the reference Kohn–Sham determinant, which in this work is chosen to be a relaxed singly excited reference. The excitation operators take the form
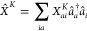
9
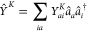
10where the indices *i*, *j* and *a*, *b* correspond to orbitals that are occupied and virtual in the reference determinant, respectively, and the amplitudes *X*_*ai*_^*K*^ and *Y*_*ai*_^*K*^ are the solutions of
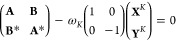
11with
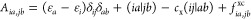
12
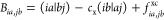
13where
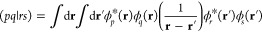
14and
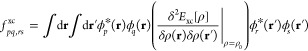
15where ϵ_*p*_ denotes the Kohn–Sham orbital eigenvalue for the single-particle orbital ϕ_*p*_, *c*_x_ controls the amount of exact exchange, and *E*_xc_ is the exchange–correlation energy. Indices *p*, *q*, *r*, and *s* denote generic orbital labels.

The transition dipole moment, μ_*KL*_ = ⟨*K*|**μ̂**|*L*⟩, can then be obtained directly as
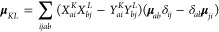
16where the electric dipole operator has the form **μ̂** = −∑_*pq*_⟨ϕ_*p*_|*e***r**|ϕ_*q*_⟩â_*p*_^†^â_*q*_ = −∑_*pq*_μ_*pq*_â_*p*_^†^â_*q*_. Note that the transition moments given by eq 16 correspond to transitions between unrelaxed TDDFT states and differ from those obtained from the quadratic response by the total neglect of the second-order relaxation of the density matrix.

Here, we demonstrate that this simplified approach yields accurate transition moments that compare well with those obtained from state-of-the-art RASSCF/RASPT2 for several multiphoton spectroscopy simulations.

### 2.3 Electronic Structure Calculations

Electronic structure calculations at both the RASSCF/RASPT2 and DFT levels were performed on the neutral PBI in the gas phase. As already mentioned, test calculations on PBI-(R^+^)_2_ give an extremely similar manifold of states (see the Supporting Information). This may be ascribed to the fact that the positive charges of the cationic species are localized on the lateral trimethylammonium moieties, that is, outside the photoactive region, which is identical in the two molecules. Simulation of PBI and PBI-(R^+^)_2_ LA is also shown to give comparable spectra (Figure S1 of the Supporting Information). For this reason, the computationally more tractable PBI is studied here.

#### 2.3.1 RASSCF/RASPT2

The PBI geometry was optimized at the MP2/ANO-L-VDZP level by imposing *D*_2*h*_ symmetry. Vibrational ground-state frequencies were computed numerically at the same level of theory. The ANO-L-VDZP basis set was also employed in the RASSCF/RASPT2 computations.

For both compounds, an active space consisting of 20 electrons in 20 orbitals was utilized and combined with a RAS protocol including up to quadruple excitations.^41^ The final SA-10-RASSCF(20,4,4;10,0,10) active space (where the first three indices denote the total number of electrons, the upper limit of holes in RAS1, and the upper limit of excitations in RAS3, respectively; while the last three indices denote the number of orbitals included in the three subspaces: in order, RAS1, RAS2, and RAS3)) is shown in Figures S2 and S3 of the Supporting Information. The single-state (SS) RASPT2 method (using the imaginary and IPEA shifts set to 0.2 and 0.0 Hartree, respectively) was employed on the SA-RASSCF wavefunction to correct the state energetics, and transition dipole moments were computed with the state-interaction method on the SA-RASSCF-optimized wavefunction. Energy gradients of the relevant (bright) excited electronic states in the Franck–Condon region were computed numerically at the (SS) RASPT2 level of theory (step size set to 0.005 Å).

The lowest 10 electronic states were considered for each of the four irreducible representations of the PBI *D*_2*h*_ point group. The full data set is listed in Table S2 of the Supporting Information, whereas energies and oscillator strengths for transitions of interest are listed in Table 1. All the multireference computations were performed with the QC software package OpenMolcas.^42,43^

#### 2.3.2 TDDFT

All DFT and TDDFT calculations employed the CAM-B3LYP exchange correlation functional and the 6-311G** basis set. The excited-state reference (S_1_) was created by promoting an electron from the HOMO to the LUMO, followed by a ground-state unrestricted DFT orbital optimization employing the maximum-overlap method.^17,18^ The S_1_ reference was then used to compute vibrational modes and frequencies and a manifold of the lowest 50 TDDFT excited states. Additional TDDFT excited-state gradient computations for all bright states within the energy window of interest were performed. All computations were carried out using a development version of the NWChem electronic structure package,^44,45^ and all TDDFT calculations employed the Tamm–Dancoff Approximation.^104^ Relevant orbitals are shown in the Supporting Information (Figure S4). State energies and oscillator strengths for the transitions of interest are listed in Table 1.

**Table 1:** RASSCF/RASPT2 and TDDFT Results for the Relevant (Spectroscopically Bright) PBI States: Labels, Symmetry, and Leading Configurations (Weights > 0.05) of the States Involved in the Transitions, as Well as Associated Energies (in eV) and Oscillator Strengths^a^

transition	state symm.	arrival state config.	weight	trans. energy	*f*
RASSCF/RASPT2					
S_0_ → S_1_	A_*g*_ → B_3*u*_	HOMO → LUMO	0.70	2.35	0.40
S_1_ → S_7_	B_3*u*_ → A_*g*_	HOMO → LUMO + 1	0.26	1.78	0.38
		HOMO ⇒ LUMO	0.07		
S_1_ → S_12_	B_3*u*_ → A_*g*_	HOMO – 2 → LUMO	0.22	2.16	0.14
		HOMO – 6 → LUMO	0.08		
		HOMO ⇒ LUMO	0.06		
		HOMO – 3 → LUMO + 2	0.06		
TDDFT					
S_0_ → S_1_	A_*g*_ → B_3*u*_	HOMO → LUMO	0.92	2.21	0.34
S_1_ → S_5_	B_3*u*_ → A_*g*_	HOMO ⇒ LUMO	0.38	1.74	0.14
		HOMO – 3 → LUMO	0.36		
		HOMO → LUMO + 1	0.21		
S_1_ → S_7_	B_3*u*_ → B_1*g*_	HOMO – 4 → LUMO	0.45	1.89	0.11
		HOMO → LUMO + 2	0.42		

a Note that the TDDFT configurations have been referred to the closed-shell configuration to facilitate the comparison with the RASSCF/RASPT2 results. The state order was assigned according to increasing energy (regardless of symmetry). Single and double arrows denote singly and doubly occupied to virtual transitions, respectively. The orbital labeling is consistent in the two sets of computations (see Figure S4 in the Supporting Information).

## 3 Experimental Techniques

TA and 2DES experiments were performed using a home-built apparatus described in detail elsewhere.^46,47^ Briefly, the setup was pumped by a Ti:Sapphire laser operating at 1 kHz and emitting 100 fs pulses centered at 800 nm. Such pulses were used to pump a noncollinear optical parametric amplifier (NOPA) that generates pulses with a spectrum spanning from 510 to 700 nm, compressed down to a sub-20 fs temporal duration by a chirped-mirror pair. For TA experiments, the NOPA output was split into two optical paths where pump and probe pulses were properly delayed. Both the pump and probe were noncollinearly focused on the sample, and the transmitted probe beam was then collected onto a high-speed spectrometer to obtain differential transmission (Δ*T*/*T*) maps as a function of the probe wavelength and probe delay. The pump fluence was set to 80 μJ/cm^2^, and the pump–probe delay was scanned up to 1000 fs with 4 fs time steps. For 2DES experiments, a sequence of three delayed pulses was needed in order to create the 2D excitation/detection maps. The first and the second pulses acted as pump pulses which were separated by a delay *t*_1_ (so-called coherence time). The third pulse acted as a probe, and it was delayed with respect to the second pulse by *t*_2_ (the so-called population time or waiting time). The nonlinear signal was measured by a spectrometer placed in the probe pulse direction, which provides the resolution of the signal along the detection frequency axis. The acquisition of a single 2DES map at a fixed *t*_2_ was obtained by Fourier-transforming the detected signal with respect to *t*_1_ for each spectral component of the probe. The result of this procedure was a 2DES map that correlates excitation and detection frequencies. This procedure was repeated for each population time *t*_2_. In this way, a sequence of 2DES maps was collected.^48^ In our apparatus, 2DES experiments were performed by introducing a birefringent delay line on the pump optical path in order to create two phase locked pulse-replicas, the translating-wedge-based identical pulses encoding system.^48^ In this way, the sequence of the two properly delayed pump-pulses was focused on the sample, together with the probe pulse, which was then collected onto the spectrometer. 2DES maps were generated by continuously scanning the coherence time from −100 to 250 fs at a fixed waiting time and by Fourier-transforming the acquired data along the coherence time *t*_1_. In this so-called partially collinear pump–probe geometry, the so-called rephasing and nonrephasing signals propagated along the same direction, allowing us to directly detect the 2DES absorptive spectra. We used the same pump fluence as for the TA experiments and we scanned the *t*_2_ delay up to 250 fs with 4 fs time steps. Frequency analysis of TA data was performed by Fourier-transforming the residual oscillations of the ΔT/T signal with respect to the waiting time at each individual detection wavelength. All the experiments were performed in a 200 μm cuvette of a solution of 1 mM PBI-(R^+^)_2_ in acetonitrile.

In the simulation of spectra, to better mimic the experimental conditions, where TA and 2DES signals were recorded by employing pulses with a finite duration and bandwidth, the simulated signals (obtained in the limit of delta-like/infinite bandwidth pulses) were filtered along the excitation frequency axis with the experimental pulse frequency–domain profile (see Figure S8 in the Supporting Information) while describing the finite time resolution by convoluting the different *t*_2_ spectra with a Gaussian function with standard deviation σ = 6.5 fs (corresponding to a FWHM of ∼15 fs).

## 4 Results and Discussion

### 4.1 Electronic Structure Method Comparison

Minor differences are observed for the optimized ground-state molecular geometries at the MP2/ANO-L-VDZP and DFT(CAM-B3LYP)/6-311G** levels of theory, with slightly shorter bond lengths (ca. 0.01/0.02 Å) exhibited at the DFT level. A comparison, at the optimized geometries, between RASSCF/RASPT2 and TDDFT for several relevant excited states—state energies, symmetry, main configurations, and oscillator strengths—is presented in Table 1. To facilitate the comparison with the RASSCF/RASPT2 results, configurations at the TDDFT level have been referred to the closed-shell configuration. The original TDDFT results (which instead refer to the HOMO → LUMO configuration) are listed in Table S1 of the Supporting Information.

Both methods describe the first excited state (of B_3*u*_ symmetry), labeled S_1_, as dominated by the HOMO → LUMO configuration (weight > 0.70) with comparable oscillator strengths. Thereby, TDDFT slightly underestimates the transition energy. Both methods predict two bright transitions out of S_1_ to higher-lying states (S_7_ and S_12_ at RASSCF/RASPT2 and S_5_ and S_7_ at TDDFT) that are expected to give rise to ESA features. Notably, the involved states exhibit a marked multiconfigurational character, dominated by configurations describing a single-electron transition with respect to the HOMO → LUMO configuration of the S_1_ state. These one-electron transitions are of three types as follows: (a) from the HOMO to LUMO, conferring a partial double excitation character to the transition; (b) from a doubly occupied orbital to the HOMO; and (c) from the LUMO to an empty virtual orbital (see Table S1). We note that the double HOMO ⇒ LUMO arrival state configuration could not be captured by standard TDDFT using the ground-state wavefunction as a reference, but it arises naturally here because an excited state (in this case S_1_) is prepared and used as the reference. The optically bright lowest excited state from S_1_ is predicted by both RASSCF/RASPT2 and TDDFT to be of A_*g*_ symmetry (labeled S_7_ and S_5_ at RAS and TDDFT levels, respectively) and to lie ca. 1.75 eV above S_1_. RASSCF/RASPT2 predicts the S_1_ → S_7_ transition to be dominated by the HOMO → LUMO + 1 configuration (corresponding to a LUMO → LUMO + 1 one-electron transition from the HOMO → LUMO reference). In contrast, TDDFT favors the double HOMO to LUMO configuration. Correspondingly, the transition exhibits different oscillator strengths, with RASSCF/RASPT2 predicting a nearly 3 times larger value. The second state that can be reached from S_1_ is much darker than the one discussed above at both levels of theory. This state has different symmetries and nature in the two calculations, and it appears in a region dominated by the S_1_ SE signal, interfering with it.

The vibrational modes displaying large coupling to the S_0_ → S_1_ electronic transition are listed in Table 2, where the strength of the coupling is presented in terms of mode-specific reorganization energies. For the transition between S_1_ and higher-lying states, only the total reorganization energy is reported. The reorganization energy is obtained projecting the excited-state gradients along the normal modes.^a^ Modes with large couplings are readily recognized by looking at the spectral densities shown in Figures S6 and S7 of the Supporting Information. In the frequency range below 1000 cm^–1^, one mode around 550/650 cm^–1^ couples strongly with the electronic structure. Interestingly, while at both levels the mode describes a symmetry-conserving breathing deformation, the motion is longitudinal at the RASSCF/RASPT2 level, whereas it is transversal at the TDDFT level. In the high-frequency range (above 1000 cm^–1^), three/four main normal modes exhibit notable couplings, all related to symmetric C=C stretching and C–H bending. The modes are depicted in Figure S5 of the Supporting Information.

**Table 2:** Top: PBI Vibrational Modes Exhibiting Notable Coupling to the S_0_ → S_1_ Transition at the RASSCF/RASPT2 and TDDFT Levels of Theory. Bottom: Total Reorganization Energies for the Spectroscopically Bright Transitions Described in Table 1^a^

RASSCF/RASPT2			TDDFT		
mode freq.	reorg. energy	S	mode freq.	reorg. energy	S
545	35	0.06	660	35	0.05
1315	160	0.12	1360	165	0.12
1480	175	0.12	1450	330	0.23
1595	200	0.12			
1620	105	0.06	1680	525	0.31

RASSCF/RASPT2		TDDFT	
transition	reorg. energy	transition	reorg. energy
S_0_ → S_1_	850	S_0_ → S_1_	1270
S_1_ → S_7_	3685	S_1_ → S_5_	2795
S_1_ → S_12_	4380	S_1_ → S_7_	1360

a The modes are labeled according to their frequency (in cm^–1^) and the strength of the electronic-nuclear coupling is presented in terms of the mode-specific reorganization energy (in cm^–1^) and Huang–Rhys factor (S).

The high-frequency modes are more strongly coupled at the TDDFT level compared to that at the RASSCF/RASPT2 level, resulting in a 50% higher total reorganization energy of the S_0_ → S_1_ transition. As we will demonstrate in the next section, this discrepancy affects the relative intensities of the vibronic bands in the linear and nonlinear spectra. In contrast, transitions to the higher-lying states exhibit stronger couplings to the vibrational modes at the RASSCF/RASPT2 level: since numerical SS-RASPT2 gradients were calculated, a possible overestimation of the coupling has to be expected when multiple states lie close in energy at the RASSCF level (due to state swapping and mixing that may occur in the energy evaluation at the displaced geometries). This issue may be alleviated by using different flavors of multireference perturbation theory, such as the multistate (MS) and the extended multistate (XMS)-RASPT2. Again, this overestimation affects the broadening and relative intensity of the ESA features in the nonlinear spectra.

### 4.2 Optical Spectroscopy

In this section, we compare the experimental data with the simulations obtained at the RASSCF/RASPT2 and TDDFT levels of theory, following the spectral simulation methodology presented in Section 2.1 and extensively discussed in ref (10). In particular, we show

- the LA spectrum;
- the TA spectra;
- 2DES maps at different waiting times *t*_2_;
- Fourier transform maps of the oscillatory component of the TA signal, aimed at resolving the vibrational dynamics.

#### 4.2.1 Linear Absorption

**Figure 2:**
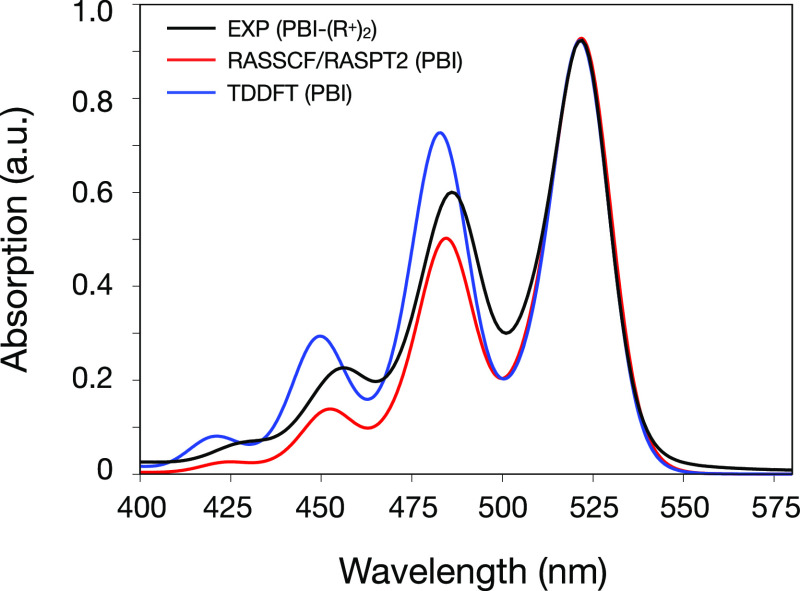
Comparison between experimental (black) and simulated—RASSCF/RASPT2 (red) and TDDFT (blue)—LA spectra. The experiments report results for the PBI-(R^+^)_2_ in acetonitrile, while the simulations were performed on the PBI neutral molecule in the gas phase. The two theoretical curves have been blue-shifted by 1200 cm^–1^ (RASSCF/RASPT2) and 2800 cm^–1^ (TDDFT), respectively, to match the experimental position of the first vibronic peak.

The LA spectrum for the neutral PBI, as compared to the experimental one for the PBI-(R^+^)_2_ monomer in acetonitrile, is shown in Figure 2. As already mentioned, test calculations on the PBI-(R^+^)_2_ were also performed, and the simulated absorption spectrum for the cationic species is shown in Figure S1 of the Supporting Information, showing negligible differences with the neutral PBI. This result is ultimately dictated by the presence of an identical photoactive region. For this reason, all the simulations of ultrafast spectroscopy were performed on the computationally cheaper neutral molecule.

Both the measured and simulated spectra display a structured spectrum in the 550–400 nm spectral range, identified as the vibronic absorption of the bright S_1_ state peaking at ca. 520, 485, 455, and 425 nm, corresponding to the fundamental (i.e., 0-0) and the first three overtones (0-1, 0-2, and 0-3). The pronounced vibronic peaks result from a few strongly coupled high-frequency normal modes described in Section 4.1. The agreement between the spectra is substantial. The simulated spectra overestimate slightly the splitting of the bands, a possible explanation being the choice not to phenomenologically downscale the normal mode frequencies as often done. Furthermore, the RASSCF/RASPT2 underestimates, whereas the TDDFT overestimates the coupling to the vibrational modes (as already observed in ref (26)), which affects the intensity of the overtones with respect to the experiment. Finally, we note that the simulated spectra have been blue-shifted by 1200 cm^–1^ (RASSCF/RASPT2) and 2800 cm^–1^ (TDDFT) to match the position of the experimental 0-0 peak. This difference may be ascribed (at least in part) to differences between the neutral PBI and the PBI-(R^+^)_2_ S_1_ state energy, the latter of which resulted being 700 cm^–1^ higher at the RASSCF/RASPT2 level (see the Supporting Information). Moreover, missing solute–solvent electrostatic and polarization effects may also affect the molecule electronic structure calculations (which were performed here in the gas phase), as highlighted in a detailed analysis in ref (26). The above-mentioned shifts have also been systematically applied to the nonlinear spectra to facilitate the comparison between experiments and simulations.

#### 4.2.2 Transient Absorption

**Figure 3:**
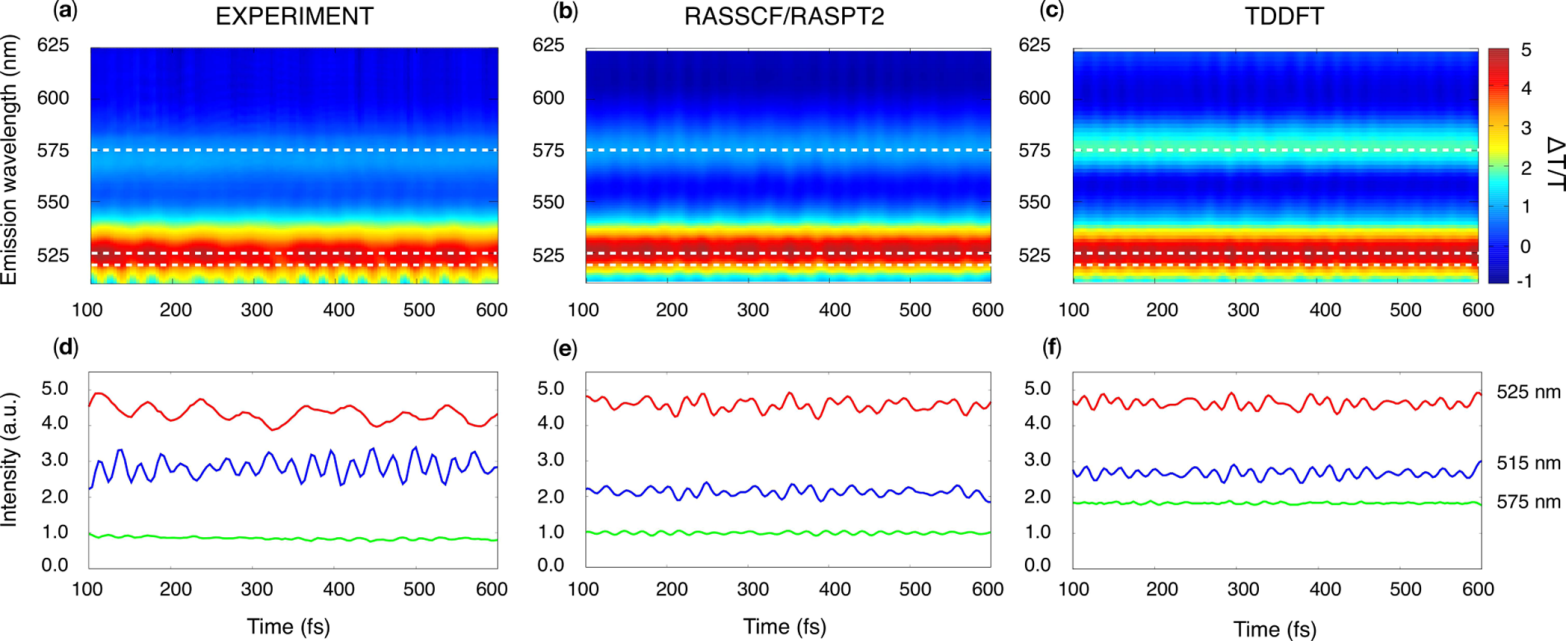
Comparison between (a) experimental and (b,c) simulated TA spectra at the RASSCF/RASPT2 and TDDFT levels of theory. (d–f) TA map cuts along specific wavelengths (namely, 515, 525, and 575 nm). The experiments report results for the PBI-(R^+^)_2_ in acetonitrile, while the simulations were performed on the PBI neutral molecule in the gas phase. The simulated spectra have been shifted [by (b) 1200 and (c) 2800 cm^–1^]. The simulated map intensity was scaled to match the experimental one, allowing us to use an identical intensity window and color range for the three maps. GSB and SE are shown in red and ESA in blue.

Figure 3 reports experimental and simulated TA maps, resolved in the 100–600 fs window after the interaction with the pump pulse. The 0–100 fs window is not shown as it is contaminated by coherent artifacts due to temporal pulse overlap.

The agreement between experimental and theoretical spectra is evident as follows: at 525 nm, one observes contributions from both the GSB and the first band of the vibronic progression of the SE, while the second band of the SE lies around 575 nm. These values also match the vibronic peak energies recorded in the steady-state emission spectrum of a PBI derivative monomer.^49^ A weak ESA feature is observed above 600 nm in a spectral region only partially covered by the experimental pulse shape. The signal labeling was facilitated by the possibility, in simulations, of selectively switching on and off contributions of different origins, which allows us to disentangle overlapping contributions and analyze their spectral dynamics separately (see Figure S9 in the Supporting Information). The overall broadening and relative intensity of the signals in the three maps are comparable. As already observed for the LA, the slight overestimation of the splitting of the vibronic bands in the simulations is reflected in the red shift of the second SE band with respect to the experiment, whereas the stronger electronic-nuclear coupling at the TDDFT level is responsible for the increased intensity of the band with respect to RASSCF/RASPT2. A more quantitative comparison can be performed by looking at cuts of the TA maps, along specific wavelengths, namely, 515, 525, and 575 nm (Figure 3d–f). One may notice that the 525 nm cut appears to be dominated by low-frequency oscillations in the experiment while containing large higher-frequency contributions in the simulations. A detailed analysis of the signal beatings is reported in the next section.

#### 4.2.3 Fourier Analysis of TA Data

**Figure 4:**
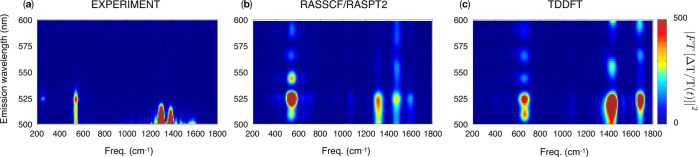
Comparison between (a) experimental and (b,c) simulated power spectra at the RASSCF/RASPT2 and TDDFT levels of theory. The experiments report results for the PBI-(R^+^)_2_ in acetonitrile, while the simulations were performed on the PBI neutral molecule in the gas phase. The simulated spectra have been shifted by 1200 and 2800 cm^–1^ along the emission wavelength axis. The simulated map maximum intensity was scaled to match the experimental one, allowing us to use an identical intensity window and color range for the three maps.

The electronic-nuclear coupling at the origin of the vibronic bands observed in the LA and TA spectra is also responsible for the intensity beating during the waiting time *t*_2_. Experimentally, the beating frequencies can be extracted by Fourier transforming the residual oscillations (i.e., the oscillatory part of the transient signal left after removing the decay trace) along the time axis, obtaining the so-called power spectrum (|*FT*[Δ*T*/*T*(*t*)]|^2^). Here, we apply the same procedure to the experimental and simulated TA traces. The power spectra, shown in Figure 4, exhibit several intense peaks whose position along the detection wavelength (*y*-axis) allows us to associate a vibrational mode with a specific spectral contribution (i.e., GSB, SE, and ESA) and thus with a specific electronic state. An analysis of the power spectra of individual contributions (i.e., SE, GSB, and GSB + SE + ESA) is shown in Figure S11 of the Supporting Information.

The beating frequencies revealed in the power spectra are consistent in the three maps and can be associated to the vibrational modes listed in Table 2. The frequency profile encompasses the breathing mode at around 540 cm^–1^ and a couple of high-frequency bands at around 1300 and 1390 cm^–1^, assigned to symmetric C=C stretching and C–H bending. We note that accounting for the finite duration of the experimental pulses is crucial in order to properly describe the relative intensity of the beating peaks in the power spectra. The finite temporal resolution sets a limit to the highest frequency detectable in the experiments below 1600 cm^–1^. If the finite resolution is not accounted for, the calculated power spectrum becomes dominated by the higher-frequency peaks due to their higher Huang–Rhys factors as listed in Table 2. A comparison between simulated maps with finite- and infinite-time resolution is shown in Figure S9 of the Supporting Information.

A few differences between experimental and simulated power spectra are worth noting. First, one observes that the peaks in the simulated maps are broader along the frequency axis. In fact, in the analysis of the experimental data, all decaying components were subtracted prior to the Fourier transform step, while in the theoretical data, this subtraction was not performed. Furthermore, one can observe that the RASSCF/RASPT2 computational level reproduces frequencies and intensities more accurately with respect to TDDFT, which gives rise to a significant blue shift of the high-frequency peak energy and overestimates the weight of the 1600 cm^–1^ mode (cf. Table 2).

Finally, the absence of peaks above 530 nm in the experimental power spectra can be ascribed to the limited bandwidth of the experimental pump pulse resonant with the 0-0 transition which suppresses to a great extent the excited-state vibrational dynamics during *t*_2_.

#### 4.2.4 2DES

**Figure 5:**
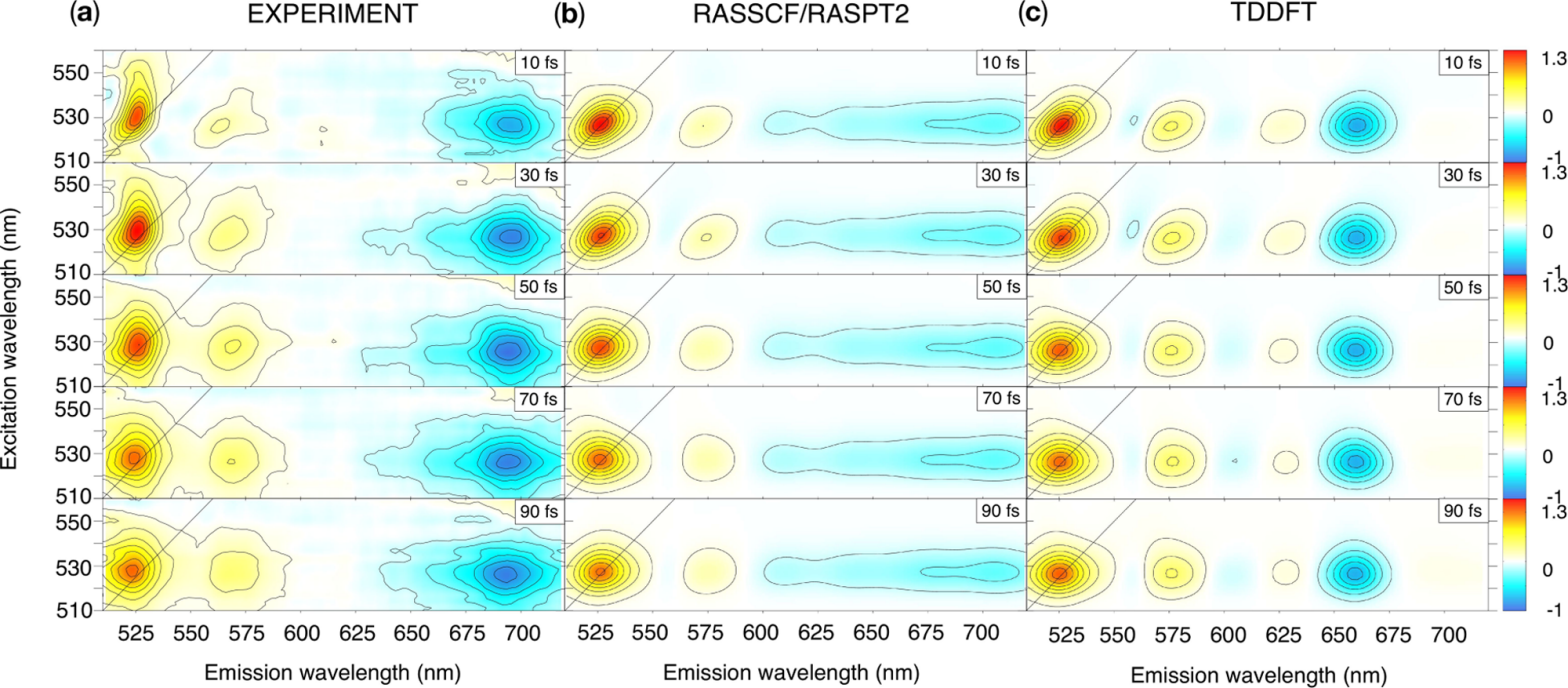
Comparison between (a) experimental and (b,c) simulated 2DES maps at few waiting times *t*_2_ at the RASSCF/RASPT2 and TDDFT levels of theory. The experiments report results for the PBI-(R^+^)_2_ in acetonitrile, while the simulations were performed on the PBI neutral molecule in the gas phase. The simulated spectra have been shifted by 1200 and 2800 cm^–1^. Their intensity was scaled to match the experimental one, allowing us to use an identical intensity window and color range for the three sets of data. GSB and SE are shown in red and ESA in blue.

2DES is capable of providing additional information regarding the time scale of spectral diffusion as it correlates the probe wavelength to the pump wavelength, effectively increasing the spectral resolution. We performed simulations of 2DES at a few selected waiting times. A comparison of the computed maps to those measured experimentally is shown in Figure 5. In the experiment, a diagonal band in the 2DES at ca. 525 nm is associated with the GSB and SE of the 0-0 line, whereas two cross-peaks from the vibronic progression of the SE are detected at ca. 570 (also seen in the TA spectrum) and 615 nm. An ESA cross-peak appears almost simultaneously after the interaction with the pump pulse pair, centered around 700 nm on the detection axis (shown in blue), extending from ca. 625 nm to the red side of the spectrum. This signal originates as a photoinduced absorption from the S_1_ state prepared by the pump pulse and can be identified with the most intense transitions listed in Table 1 (i.e., S_1_ → S_7_ at the RAS level or S_1_ → S_5_ at the TDDFT level). Note that a different pulse shape was employed here with respect to the previously reported TA spectrum; such a pulse shape extends the probe bandwidth in the red to 750 nm allowing us to clearly capture the photoinduced absorption, whose weak tail was only faintly visible in the TA spectrum (see Figure S8 in the Supporting Information).

The main experimental peaks are successfully reproduced by the simulations; however, some discrepancies are apparent. The RASSCF/RASPT2 ESA peak appears strongly broadened, spreading in the range 600–750 nm. As a consequence, it is also less intense. This spread can be rationalized in part by the large reorganization energy found for state S_7_ (at the RAS level or S_5_ at the TDDFT level, see Table 1), which may be overestimated in numerical SS-RASPT2 gradient calculations, and also partially by the neglect of the finite pulse bandwidth in the simulation. A refined treatment of the limited experimental bandwidth, in resonance almost exclusively with the first vibronic band of the absorption, would produce a cold wave-packet at the bottom of the S_1_ well. In contrast, the δ-pulse used in the present simulations leads to an excess of kinetic energy of the wave-packet, which eventually broadens the ESA peak. Note how, in the case of the RASSCF/RASPT2 results, this broadened ESA peak partially covers the third peak of the S_1_ SE vibronic progression, which is instead clearly visible around 615 and 625 nm in the experimental and TDDFT maps, respectively. Note also that this peak is much stronger in the TDDFT maps than in the experiment due to the overestimation of the S_0_ → S_1_ vibronic coupling at the TDDFT level.

**Figure 6:**
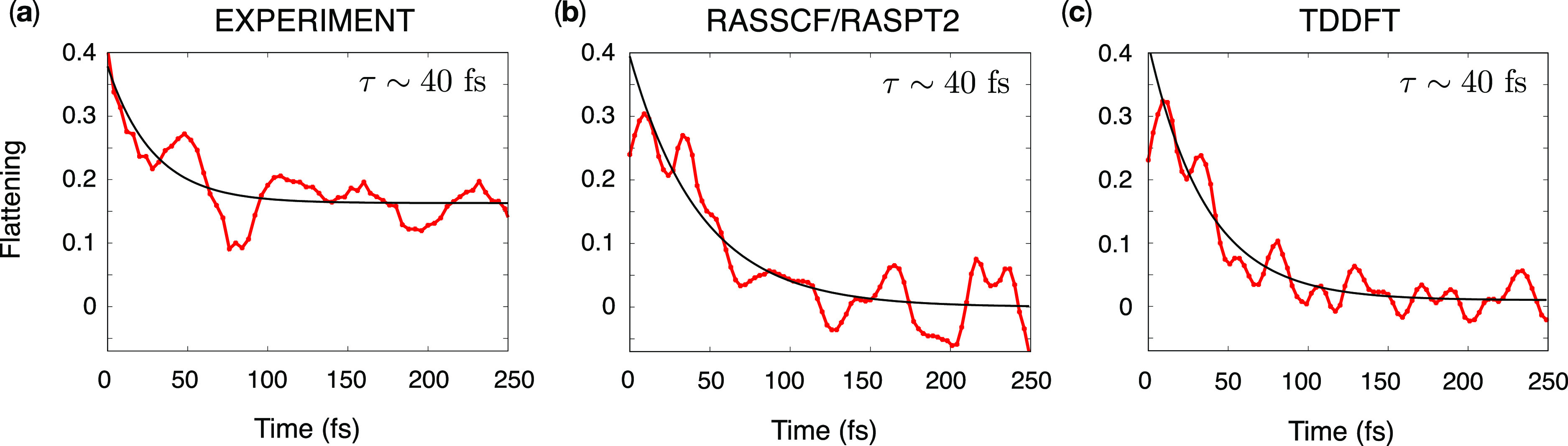
Comparison between (a) experimental and (b,c) simulated spectral diffusion as captured by the flattening parameter (i.e., the difference between the diagonal and antidiagonal widths of the 525 nm peak in the 2DES, normalized to the diagonal width) along the *t*_2_ time. The value of the flattening is shown in red, while the fit with an exponential is shown in black. The decay time τ is also shown.

Regarding the signal dynamics along *t*_2_, we observe a marked spectral diffusion, characterized by the progressive circularization (i.e., loss of correlation between the pump and probe) of the initially elongated peaks at 525 and 575 nm emission wavelengths following the memory loss in the system due to the solvent reorganization around the PBI molecule. This effect can be captured by a number of quantitative measures (flattening, ellipticity, central line slope, etc.).^50^ Here, we employed the flattening parameter, obtained as the difference between the diagonal and antidiagonal widths of a peak, normalized to the diagonal width. The evolution of the flattening parameter for the diagonal peak as a function of the waiting time *t*_2_ is shown in Figure 6 for both experiments (a) and simulations (b,c). Note that the initial flattening value is larger in the experiment as the experimental peak is tilted more strongly at early times. An exponential fit of the decay of the flattening provides the time scale τ of the solvent reorganization, being ca. 40 fs in theory and experiment. As the simulations were performed in the gas phase, this phenomenon was introduced through the OBO spectral density whose time-scale parameter Λ^–1^ was chosen precisely as 40 fs (see Section 2.1) to reproduce the fastest experimental time scale. This value can also be obtained evaluating the solvent-induced energy gap fluctuation along a molecular dynamics simulation.^51^ Notably, the experimental flattening does not decay to zero at longer times (250 fs) at variance with the simulations. This suggests the presence of a second, slower relaxation time scale, which was not modeled in the simulations. Note that the presence of a fast and slow time scale for the acetonitrile relaxation dynamics has been reported in the literature in both theoretical and experimental studies.^105,106^ Finally, the flattening is observed to oscillate along the *t*_2_ time, and the Fourier analysis of these oscillations returns the same intramolecular beating frequencies discussed above.

## 5 Conclusions

This paper demonstrates the power of in silico spectroscopy to reproduce, interpret, and, in some cases, predict experimental ultrafast optical spectroscopy data. We reported the simulation of linear and nonlinear spectroscopy of the neutral PBI at two levels of theory, namely, RASSCF/RASPT2 and TDDFT. The obtained results were compared with experiments performed in acetonitrile.

While the RASSCF/RASPT2 approach is well-known for being capable of simulating nonlinear spectroscopy from first principles,^8,12−14^ the present work shows that, in principle, a TDDFT protocol can be also adapted for the same purposes. The calculated PBI spectra at both levels of theory show a remarkable agreement with the experimental data. These include LA (which probes the vibronic structure of the first singlet excited state, S_1_) and nonlinear techniques such as TA and 2DES (for which a reliable description of higher-lying states is crucial). Both static (signals positions and intensities) and dynamical spectral properties (such as intensity beating and spectral diffusion) are reproduced to a satisfactory agreement. The microscopic origin of the main spectral features of PBI in solution is demonstrated as follows: the beating frequencies are assigned to specific molecular Raman-active modes. The importance of incorporating the finite time resolution and limited bandwidth of the experiment in the simulations is also discussed.

A detailed comparison of the QC sets of data obtained at the two levels of theory was carried out, demonstrating that TDDFT with a relaxed singly excited configuration as reference succeeds in capturing states with a doubly excited-state character, elusive to standard (ground state) TDDFT. Moreover, dipolar couplings between excited states can be computed straightforwardly (within a pseudowavefunction representation), thereby rendering accessible all the quantities required to simulate nonlinear spectra.

We note that the TDDFT approach may encounter convergence issues when the reference state is not dominated by single configurations. Furthermore, it remains blind to high-lying states described through multiple excitations out of the reference state (i.e., states dominated by triple- or higher-order excitations), which nonetheless will in general produce negligible contributions in the simulated spectra. Photoexcited molecules evolving to states of doubly excited nature will instead continue to pose a challenge. Future work in this direction will be carried out to demonstrate the performance of the method and its range of applicability in diverse molecular systems.

## Supporting Information Available

Summary of the parameters employed in the spectroscopy simulations; configurations of TDDFT states; PBI and PBI-(R^+^)_2_ LA spectra, RASSCF active space orbitals, and excited-state energies; PBI-relevant DFT orbitals; normal modes at the MP2 and DFT levels of theory; spectral densities of the bright transitions; shape of the experimental laser pulses; TA spectrum for GSB, SE, and GSB + SE contributions; Fourier analysis of TA data with finite and infinite resolution; Fourier analysis of TA data for GSB, SE, and GSB + SE contributions; and cuts of the 2DES spectra at selected *t*_2_ times (PDF)

## Footnotes

a The coupling with modes below 300 cm^–1^ was set to zero.
