# Supplementary material for: In Silico Ultrafast Nonlinear Spectroscopy Meets Experiments: The Case of Perylene Bisimide Dye
Source: J Chem Theory Comput. 2021 Oct 22;17(11):7134–45. doi: 10.1021/acs.jctc.1c00570 (PMC8582250; doi:10.1021/acs.jctc.1c00570)
Supplement: Supplementary file 1 — ct1c00570_si_001.pdf [file ct1c00570_si_001.pdf]

## Supporting Information

for

# In silico ultrafast nonlinear spectroscopy meets experiments: the case of perylene bisimide dye


Francesco Segatta,[*,†] Mattia Russo,[‡] Daniel R. Nascimento,[¶,⊥] Davide Presti,[†] Francesco Rigodanza,[§] Artur Nenov,[†] Andrea Bonvicini,[†] Alberto Arcioni,[†] Shaul Mukamel,[‖] Margherita Maiuri,[‡] Luca Muccioli,[†] Niranjan Govind,[*,¶] Giulio Cerullo,[*,‡] and Marco Garavelli[*,†]

†*Dipartimento di Chimica Industriale "Toso Montanari", Università di Bologna, Viale del Risorgimento 4, I-40136 Bologna, Italy*

‡*IFN-CNR, Dipartimento di Fisica, Politecnico di Milano, P. Leonardo da Vinci 32, I-20133 Milan, Italy*

¶*Physical and Computational Sciences Directorate, Pacific Northwest National Laboratory, Richland, Washington 99352, USA*

§*Dipartimento di Scienze Chimiche, Università degli studi di Padova, Via F. Marzolo, I - 35131 Padova, Italy*

‖*Department of Chemistry and Department of Physics and Astronomy, University of California, Irvine, 92697, USA*

⊥*Department of Chemistry, The University of Memphis, Memphis, Tennessee 38152, USA*

E-mail: francesco.segatta2@unibo.it; niri.govind@pnnl.gov; giulio.cerullo@polimi.it; marco.garavelli@unibo.it

# This PDF includes:

- Summary of the parameters employed in the spectroscopy simulations;
- Configurations of TDDFT states: comparison of different representations;
- PBI and PBI-$(R^+)_2$ linear absorption spectra;
- PBI and PBI-$(R^+)_2$: RASSCF active space orbitals and excited state energies;
- PBI relevant DFT molecular orbitals;
- Normal modes at the MP2 and DFT level of theory;
- Spectral densities of the bright transitions;
- Shape of the experimental laser pulses and their overlap with the (experimental) linear absorption spectrum;
- TA spectrum for GSB, SE, and GSB+SE contributions;
- Fourier Analysis of TA data with finite and infinite resolution;
- Fourier Analysis of TA data for GSB, SE, and GSB+SE contributions;
- Cuts of the 2DES spectra at selected $t_2$ times.

# S1 Summary of the parameters employed in the spectroscopy simulations

Simulations were carried on with $t_2$ in the interval $(0, 600)$ fs. In fact, the relevant dynamics happens before 600 fs, and 600 fs upper limit is long enough to safely perform the Fourier transform of the low frequency modes.

The time delay between consecutive $t_2$ points was set to 4 fs. This is short enough to allow proper sampling of the high-frequency modes (so that they can be revealed by Fourier transform of the data), but also long enough to reduce the number of $t_2$ snapshots to be evaluated.

Other parameters include the OBO $\lambda$ and $\Lambda^{-1}$ values, set to 240 cm$^{-1}$ and 40 fs respectively, and whose meaning has been explained in the main text.

As discussed in the main text, the lifetime of the $S_1$ state was set to infinity (i.e. very long compared to the time scale of the simulations reported here). This allows to use analytic expressions for the response functions.

# S2 Configurations of TDDFT states: comparison of different representations

The following Table reports on the leading configuration (weight $> 0.05$) of the states involved in the bright transitions. Two different representations are compared: the one directly obtained from the calculations (in which the HOMO$\rightarrow$LUMO configuration is used as a reference), and one in which the closed shell GS configuration is used as a reference. The latter was also employed in Table 1 of the main text, as it makes the comparison with RASSCF/RASPT2 simpler. To make an example, the HOMO $\rightarrow$ LUMO transition from the HOMO $\rightarrow$ LUMO reference, corresponds to a HOMO $\Rightarrow$ LUMO from the closed-shell reference; the HOMO$-$3 $\rightarrow$ HOMO transition from the HOMO $\rightarrow$ LUMO reference, instead, corresponds to a HOMO$-$3 $\rightarrow$ LUMO transition.

Table S1: Comparison of TDDFT leading configurations (weights $> 0.06$) of the states involved in the transitions, for a) the GS (closed-shell configuration) as reference, and b) the $S_1$ state (HOMO $\rightarrow$ LUMO configuration) as reference. Single and double arrows denote single and double occupied to virtual transitions, respectively. The orbital are labeled consistently to their depiction if Figure S4.

| | | TDDFT | | |
|---|---|---|---|---|
| Transition | State Symm. | Arrival St. Config. (GS ref.) | Arrival St. Config. ($S_1$ ref.) | Weight |
| $S_0 \rightarrow S_1$ | $A_g \rightarrow B_{3u}$ | HOMO $\rightarrow$ LUMO | - | 0.92 |
| $S_1 \rightarrow S_5$ | $B_{3u} \rightarrow A_g$ | HOMO $\Rightarrow$ LUMO | HOMO $\rightarrow$ LUMO | 0.38 |
| | | HOMO$-$3 $\rightarrow$ LUMO | HOMO$-$3 $\rightarrow$ HOMO | 0.36 |
| | | HOMO $\rightarrow$ LUMO+1 | LUMO $\rightarrow$ LUMO+1 | 0.21 |
| $S_1 \rightarrow S_7$ | $B_{3u} \rightarrow B_{1g}$ | HOMO$-$4 $\rightarrow$ LUMO | HOMO$-$4 $\rightarrow$ HOMO | 0.45 |
| | | HOMO $\rightarrow$ LUMO+2 | LUMO $\rightarrow$ LUMO+2 | 0.42 |

## S3 PBI and PBI-$(R^+)_2$ linear absorption spectra

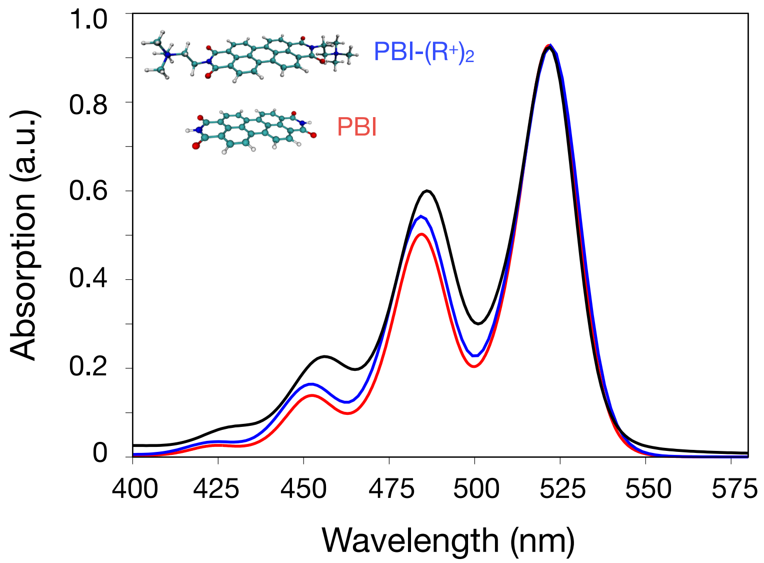


Figure S1: Linear absorption (vibronic) spectra of PBI (red) and PBI-$(R^+)_2$ ion (blue) computed at RASSCF/RASPT2 level of theory; due to the computational cost of the MP2 method, frequencies has been computed at DFT level (using $\omega$B97X-D functional and 6-31G** basis-set). To match the experimental $\lambda_{max}$ of a 1 mM solution of PBI-$(R^+)_2$ in acetonitrile (black curve), the computed spectra have been blue-shifted by $+1200$ cm$^{-1}$ (PBI) and $+400$ cm$^{-1}$ (PBI-$(R^+)_2$). Upon normalization to the maximum of the first vibronic band, the successive vibronic bands of PBI-$(R^+)_2$ ion are more intense with respect to those of the neutral PBI and closer to the experimental spectra. The total reorganization energy for the $S_1$ state in PBI-$(R^+)_2$ is 960 cm$^{-1}$.

## S4 PBI and PBI-$(R^+)_2$: RASSCF active space orbitals

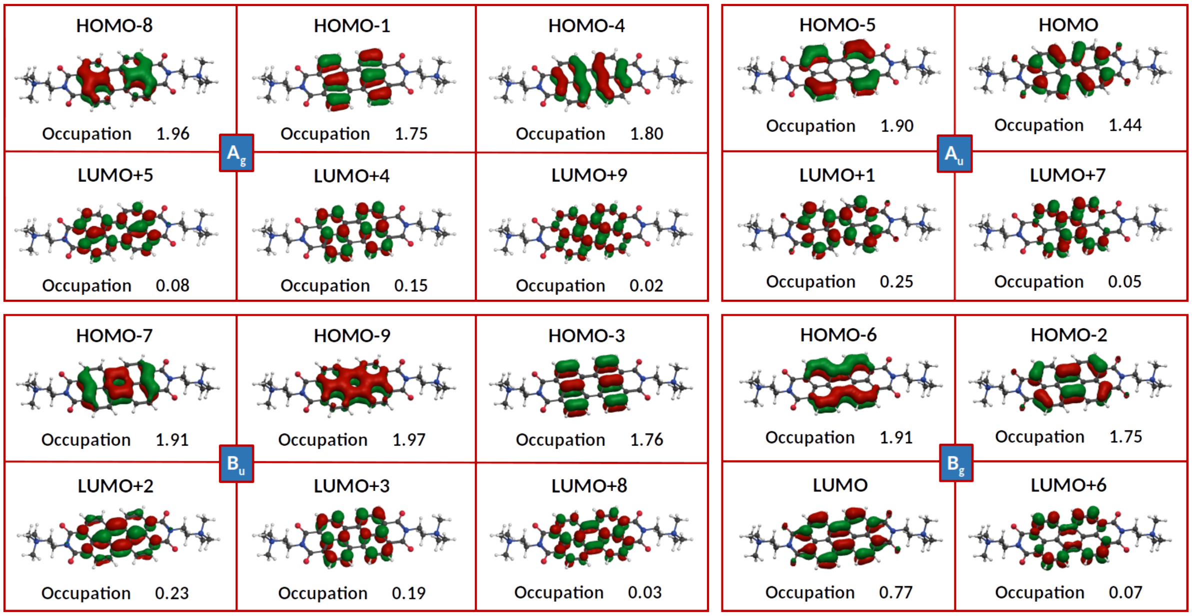


Figure S2: SA-10-RAS(20,4,4;10,0,10) natural orbitals used for multireference calculations on PBI-$(R^+)_2$ ion. Natural orbitals are grouped according to the irreducible representations of $C_{2h}$ symmetry point group.

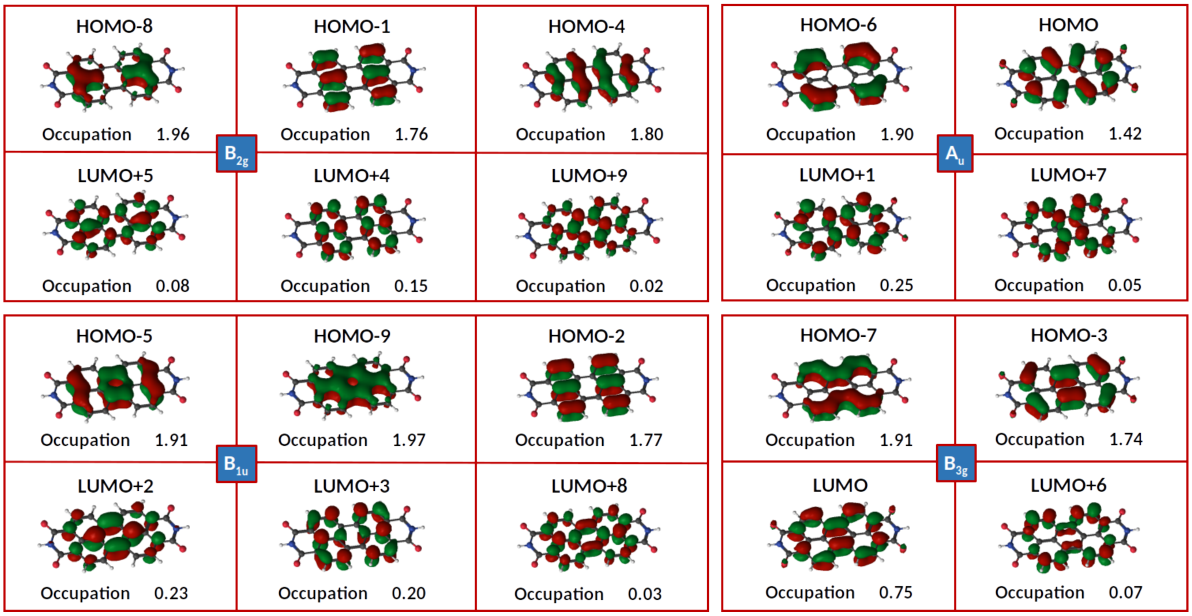


Figure S3: SA-10-RAS(20,4,4;10,0,10) natural orbitals used for multireference calculations on PBI (neutral) molecule. Molecular orbitals are grouped according to the irreducible representations of $D_{2h}$ symmetry point group.

## S5 PBI and PBI-$(R^+)_2$: RASPT2 excited state energies

Table S2: The lowest ten electronic states were considered for all allowed symmetries (four) of the $C_{2h}$ and $D_{2h}$ point groups of **PBI** and **PBI-$(\mathbf{R}^+)_2$**. Here we report the states transition energy at the SS-RASPT2 level. The **PBI** $S_0$, $S_1$, $S_7$ and $S_{12}$ and the **PBI-$(\mathbf{R}^+)_2$** $S_0$ and $S_1$ states are highlighted in bold. Transition dipole moments were computed for relevant transitions allowed by symmetry, i.e. $A_g \rightarrow B_{2u}/B_{3u}$ and $B_{3u} \rightarrow A_g/B_{1g}$ for PBI, and $A_g \rightarrow A_u/B_u$ and $B_u \rightarrow A_g/B_g$ for PBI-$(R^+)_2$. The brightest transitions have been reported in the main text.

| | **PBI** | | | | **PBI-$(\mathbf{R}^+)_2$** | | | |
|---|---|---|---|---|---|---|---|---|
| State | $A_g$ (eV) | $B_{3u}$ (eV) | $B_{2u}$ (eV) | $B_{1g}$ (eV) | $A_g$ (eV) | $B_g$ (eV) | $A_u$ (eV) | $B_u$ (eV) |
| 1 | **0.00** ($S_0$) | **2.35** ($S_1$) | 3.31 | 3.07 | **0.00** ($S_0$) | 3.18 | 3.41 | **2.45** ($S_1$) |
| 2 | 3.08 | 4.52 | 4.48 | 3.40 | 3.32 | 3.51 | 4.67 | 4.69 |
| 3 | **4.13** ($S_7$) | 4.84 | 4.64 | 3.48 | 4.06 | 3.58 | 4.73 | 4.91 |
| 4 | 4.44 | 5.41 | 5.01 | 4.29 | 4.75 | 4.20 | 5.03 | 5.60 |
| 5 | **4.51** ($S_{12}$) | 5.28 | 4.48 | 4.63 | 4.59 | 4.91 | 4.66 | 5.32 |
| 6 | 5.16 | 5.66 | 5.21 | 5.55 | 5.35 | 5.73 | 5.31 | 5.61 |
| 7 | 5.47 | 6.15 | 5.67 | 5.92 | 5.58 | 6.11 | 5.83 | 6.08 |
| 8 | 5.87 | 5.68 | 5.53 | 5.83 | 6.07 | 5.91 | 5.70 | 5.93 |
| 9 | 5.99 | 5.57 | 6.62 | 5.91 | 6.16 | 6.14 | 6.82 | 5.60 |
| 10 | 6.15 | 6.45 | 6.09 | 6.56 | 6.28 | 6.63 | 5.93 | 6.73 |

# S6 PBI relevant DFT molecular orbitals

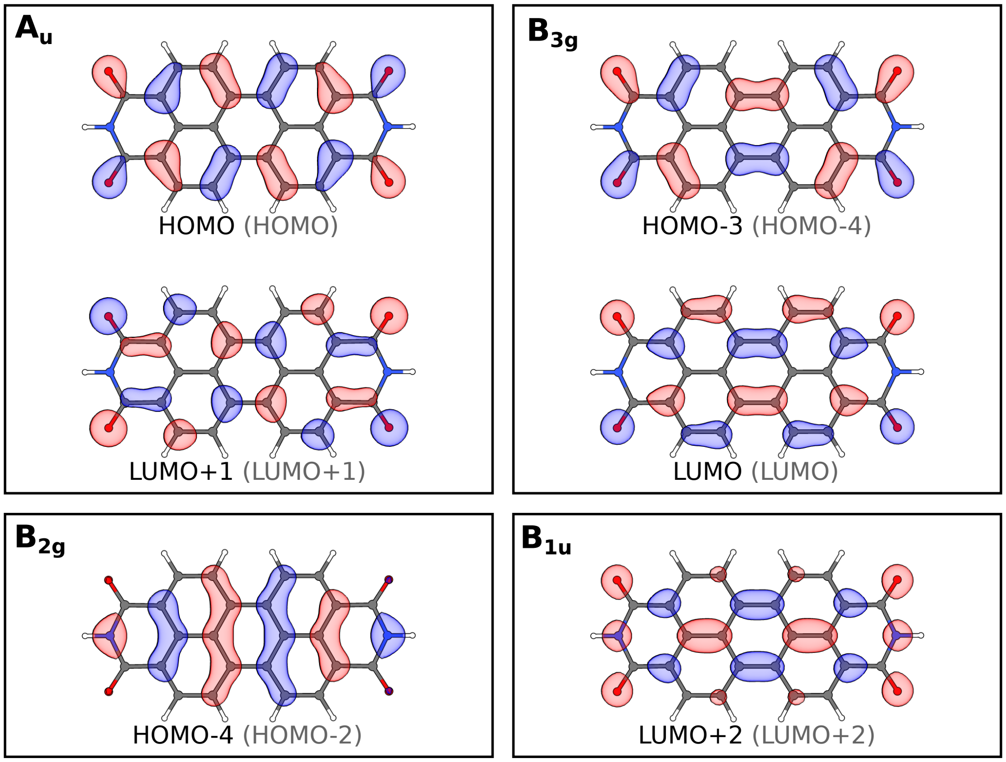


Figure S4: Relevant DFT molecular orbitals for $S_1$, $S_5$ and $S_7$ excited states of PBI (isovalue 0.01). Molecular orbitals are grouped according to the irreducible representations of $D_{2h}$ point group. The orbitals are labeled in two ways: the black label below each molecular orbital refers to the RASSCF assignation of Figure S3 (which facilitate the comparison of the states configurations of Table 1 in the main text), while the grey label in parentheses refers to the original DFT ordering.

# S7 Analysis of normal modes and frequencies

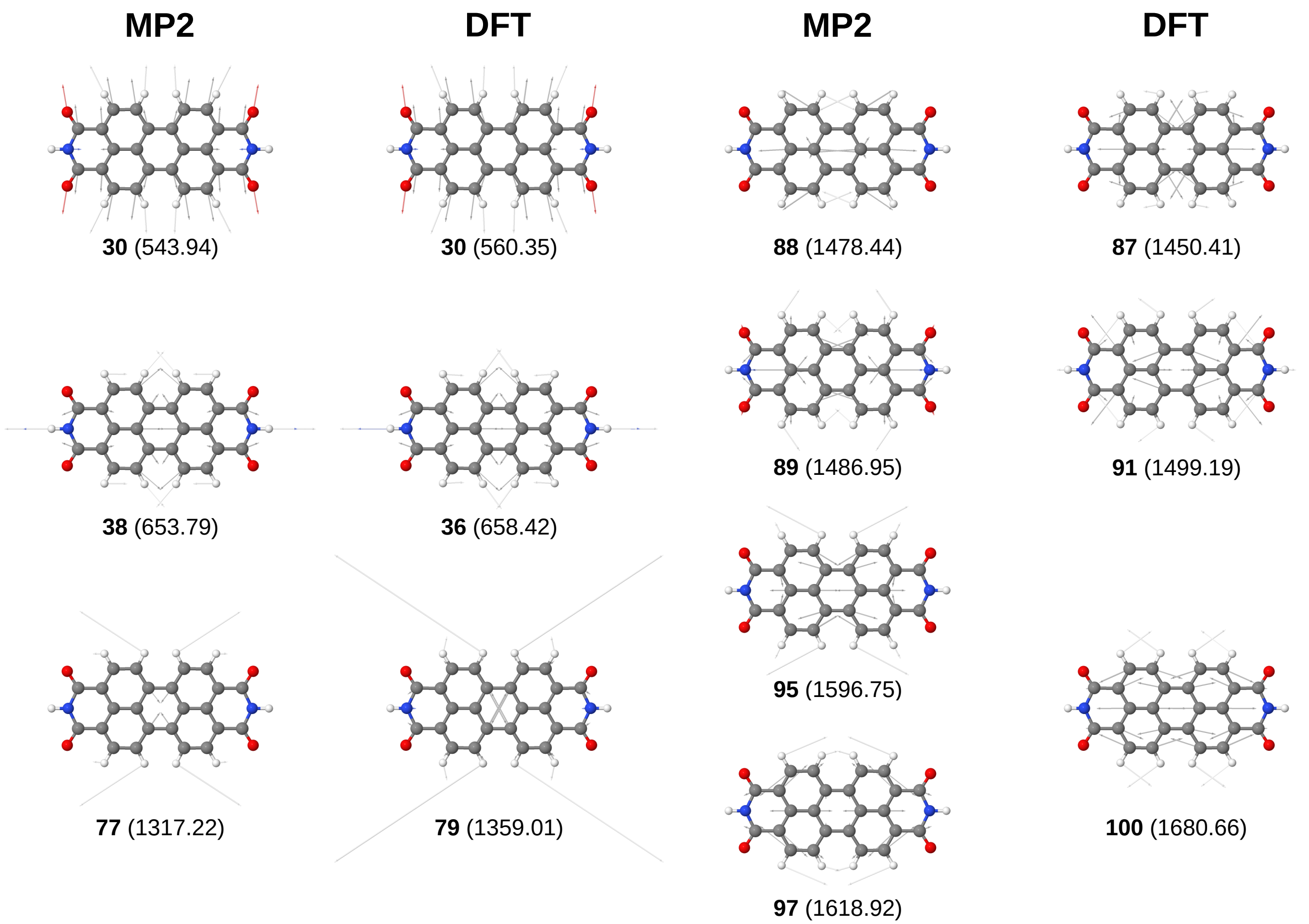


Figure S5: Selected normal modes computed at MP2 and DFT level of theory. For each normal mode, the corresponding frequency (in $cm^{-1}$) is also reported in parenthesis. All normal modes reported here are of $A_g$ symmetry (i.e. total symmetric modes).

## S8 Spectral densities

### S8.1 $J(\omega)$ for the $S_1$ state

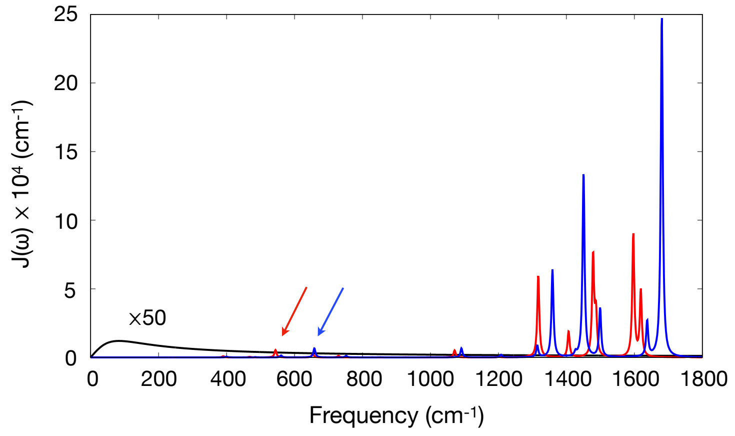


Figure S6: Spectral densities for the $S_0 \rightarrow S_1$ transition at TDDFT (blue) and RASSCF/RASPT2 (red) level of theory. The arrows highlight the PBI breathing modes that are responsible for the low frequency beatings observed in the power spectrum. The over-damped brownian oscillator spectral density ($J_{OBO}(\omega)$) with $\lambda = 240$ cm$^{-1}$ and $\Lambda^{-1} = 40$ fs is also reported (black line).

### S8.2 $J(\omega)$ for the $S_7$ ($S_5$) RASSCF/RASPT2 (TDDFT) state

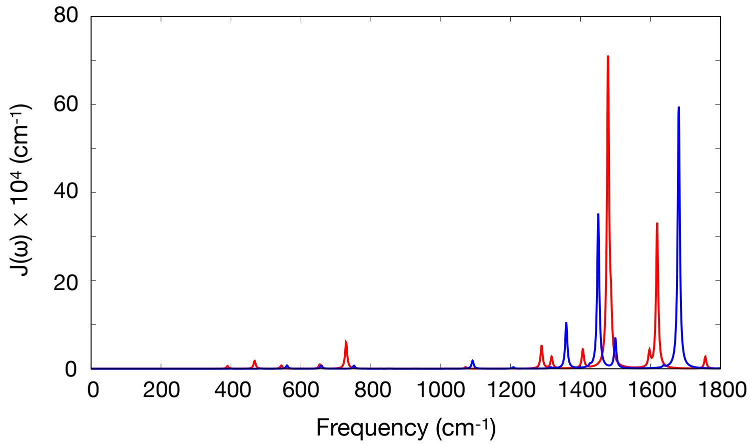


Figure S7: Spectral densities for the transitions that gives rise to the brightest ESA signal in the nonlinear spectra, namely: $S_1 \rightarrow S_5$ at TDDFT level (blue line), and $S_1 \rightarrow S_7$ at RASSCF/RASPT2 level (red line).

## S9 Linear absorption and pulse shapes

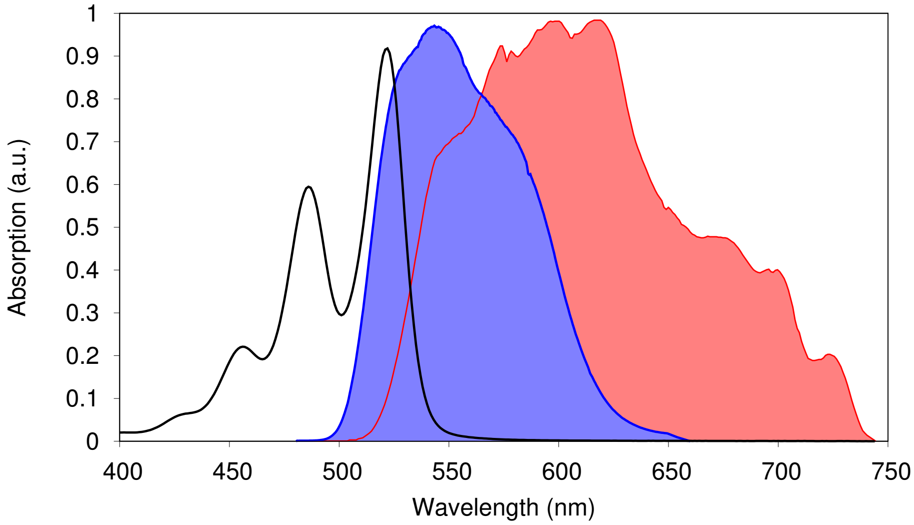


Figure S8: Experimental linear absorption spectrum of PBI (black) and pulse shapes used in the experiments. The blue pulse shape was used for TA, while the red pulse shape was employed in 2DES experiments. Note how the latter covers a region in which the ESA signals are observed, therefore allowing for their detection.

# S10 Transient Absorption selected contributions

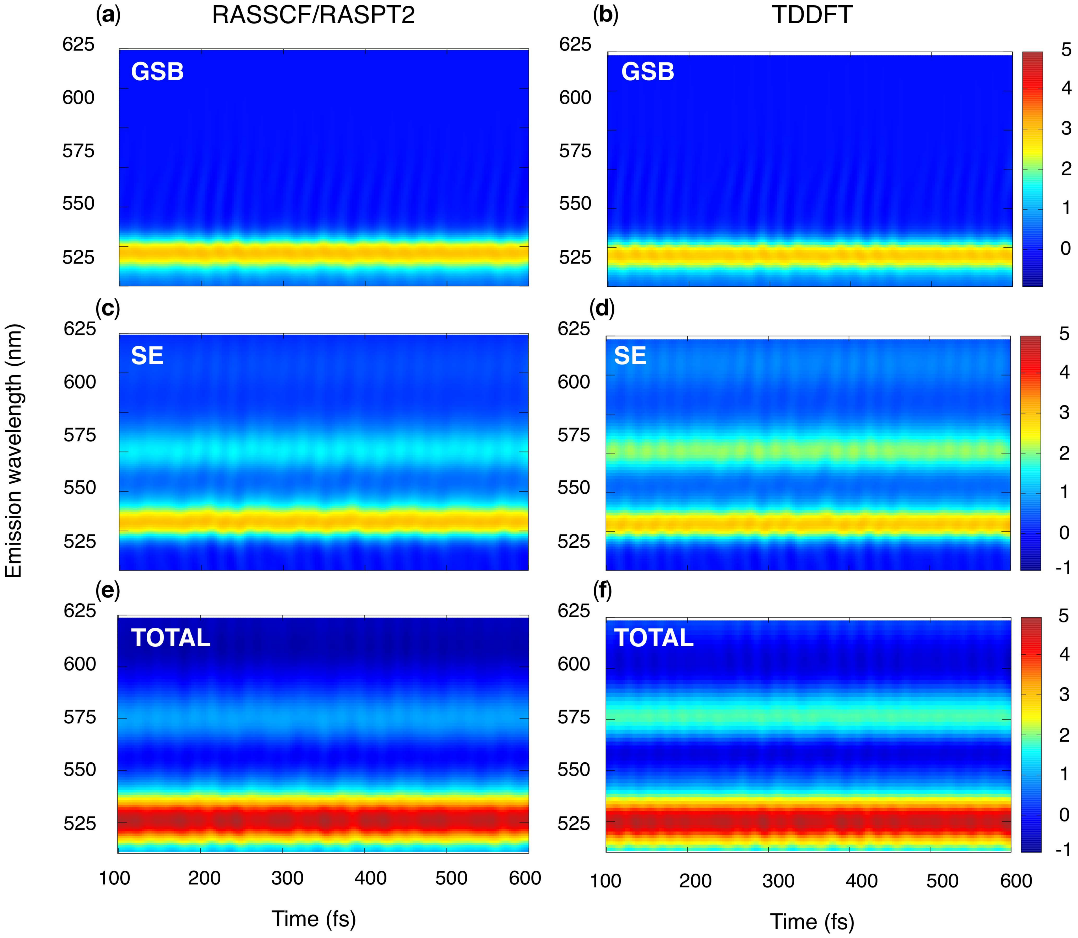


Figure S9: (a,b) GSB, (c,d) SE and (e,f) TOTAL TA maps at both RASSCF/RASPT2 and TDDFT levels of theory. The ability of selectively switching on and off different contributions of the total TA map, allows to untangle and label the origin of the observed signals, also in regions where these may overlap (as it happens around 525 nm).

## S11 Fourier Analysis with infinite and finite time resolution

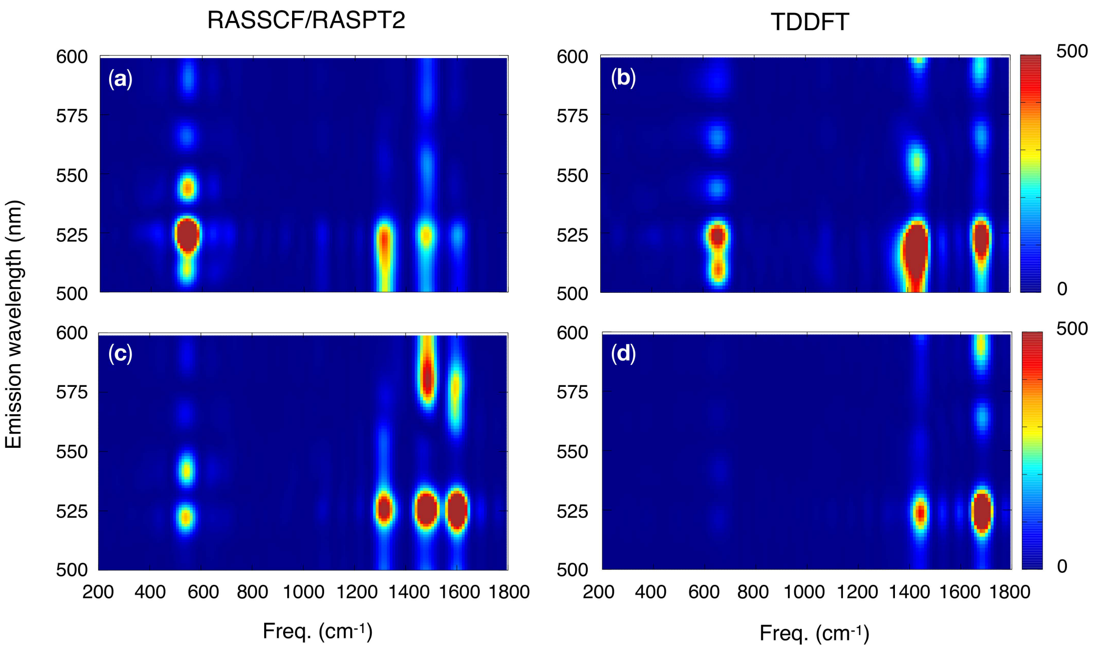


Figure S10: Fourier analysis of the TA maps at the two levels of theory, with finite (a-b) and infinite (c-d) time resolution. Note that the high frequency modes appear quenched when (realistic) finite resolution effects are included. The longer the pulse width in time, the lower the resolution, the larger the quenching of the high-frequency beatings peaks is.

## S12 Fourier Analysis of TA selected contributions

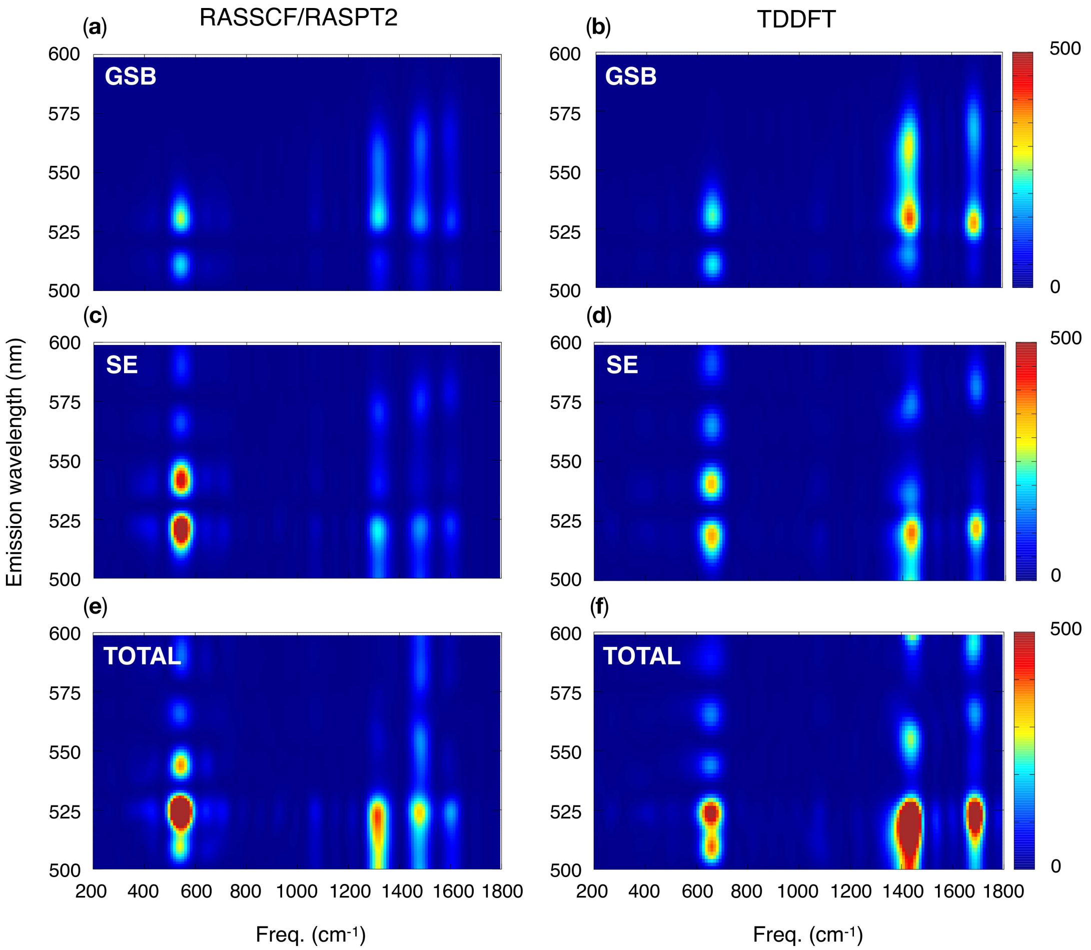


Figure S11: (a,b) GSB, (c,d) SE and (e,f) TOTAL Fourier transformed TA maps at both RASSCF/RASPT2 and TDDFT levels of theory. Note that the GSB and SE contributions give rise to peaks in different regions of the map. Note also the different position of the horizontal nodes, located at ca. 520 and 530 nm in the GSB and SE maps, respectively, i.e. at the maxima of the absorption and emission spectra.

# S13 Cuts of the 2DES spectra at selected $t_2$ times

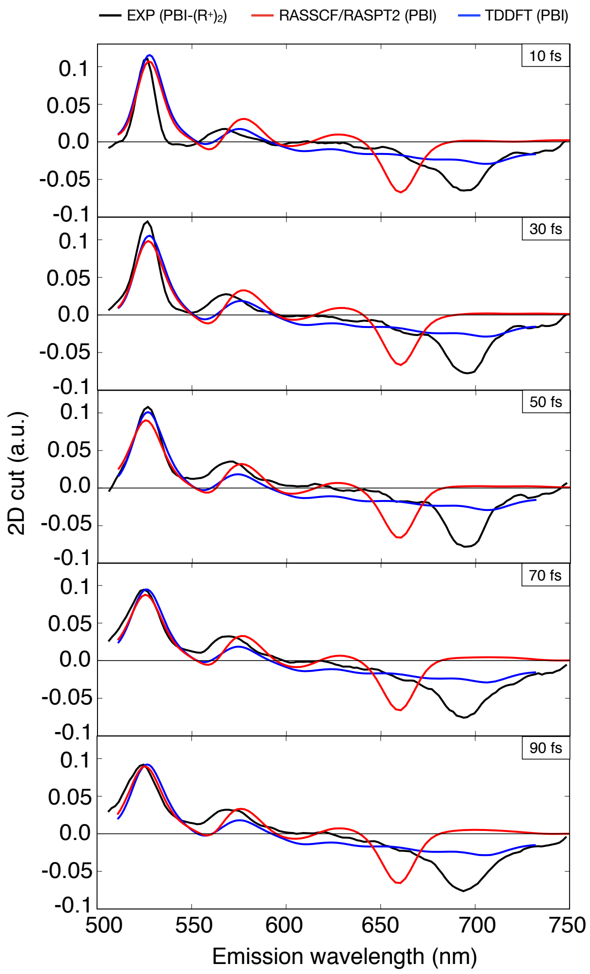


Figure S12: Comparison of some cuts of the 2DES maps reported in Figure 5 of the main text, for both experiment and simulations. The cuts were performed at Excitation wavelength equal to 530 nm, and for the $t_2$ times 10, 30, 50, 70 and 90 fs. The main qualitative features of the experiment are reproduced in the simulations, while quantitative differences can be noticed in terms of peak position (minor red-shift of the SE progression at both levels of theory, and blue-shift of the ESA peak at the TDDFT level) and line-shape (excess of broadening of the main GSB/SE peak at early times at both levels of theory, and for the ESA peak at all times at the RASSCF/RASPT2 level).
